# Gene-enhanced tissue engineering for dental hard tissue regeneration: (2) dentin-pulp and periodontal regeneration

**DOI:** 10.1186/1746-160X-2-16

**Published:** 2006-05-25

**Authors:** Paul C Edwards, James M Mason

**Affiliations:** 1 Creighton University School of Dentistry, Omaha, NE, USA; 2 NorthShore- Long Island JewishFeinstein Institute for Medical Research, Manhasset, NY, USA

## Abstract

Potential applications for gene-based tissue engineering therapies in the oral and maxillofacial complex include the delivery of growth factors for periodontal regeneration, pulp capping/dentin regeneration, and bone grafting of large osseous defects in dental and craniofacial reconstruction.

Part 1 reviewed the principals of gene-enhanced tissue engineering and the techniques of introducing DNA into cells. This manuscript will review recent advances in gene-based therapies for dental hard tissue regeneration, specifically as it pertains to dentin regeneration/pulp capping and periodontal regeneration.

## i. Introduction

The goal of gene-enhanced tissue engineering is to regenerate lost tissue by the local delivery of cells that have been genetically-enhanced to deliver physiologic levels of specific growth factors. The basis for this approach lies in the presence of a population of progenitor cells that can be induced, under the influence of these growth factors, to differentiate into the specific cells required for tissue regeneration, with guidance from local cues in the wound environment [1].

From a tissue engineering approach, the oral cavity has significant advantages compared to other sites in the body, including easy access and observability. Potential applications for gene-based tissue engineering therapies in the oral and maxillofacial complex include the delivery of growth factors for periodontal regeneration, pulp capping/dentin regeneration, treatment of malignant neoplasms of the head and neck [2], regeneration for bone grafting of large osseous defects in dental and craniofacial reconstruction (e.g. bone augmentation prior to prosthetic reconstruction, fracture repair, and repair of facial bone defects secondary to trauma, tumor resection, or congenital deformities), and articular cartilage repair [3,4].

This manuscript will review recent advances in gene-based therapies for dental hard tissue regeneration, specifically as it pertains to dentin regeneration/pulp capping and periodontal regeneration.

## ii. Gene-based therapies for dentin/pulp regeneration

### A. Background

The goal of modern restorative dentistry is to functionally and cosmetically restore lost tooth structure. Destroyed coronal tooth structure, most commonly resulting from dental caries, is currently restored using metal or polymer-based materials; primarily silver amalgam, resin-based composites and metal or porcelain crowns. Although these conventional restorative materials have proven to be highly effective at preserving teeth, they have a limited life-span and ultimately require replacement. It is estimated that in the United States alone, close to 200 million restorations, or 2/3 of all restorations placed by dentists, involve the replacement of failed restorations [5]. Moreover, a significant percentage of these restored teeth ultimately undergo pulpal necrosis, requiring either tooth extraction or endodontic treatment and prosthetic buildup. Therefore, development of novel techniques to regenerate, as opposed to repairing, lost tooth structure would have significant benefits.

Potential applicability of any dental hard tissue regenerative protocol could include the regeneration of an entire missing tooth or the regeneration of specific components of an otherwise viable tooth (e.g. a decayed tooth with early pulpal involvement). The lack of any enamel forming cells in the enamel of fully developed erupted teeth precludes the potential for cell-based approaches for enamel regeneration.

In contrast, the regeneration of dentin is feasible because dentin is in intimate contact with an underlying highly vascular and innervated pulpal tissue, forming a tightly-regulated "dentin-pulp complex". During primary tooth formation, dentin is produced by odontoblastic cells located within the pulp. Following tooth eruption, the secretory activity of these cells is down-regulated, although they continue to produce secondary dentine at a low level. Pulpal tissue retains a limited potential to repair itself following various insults. These healing stages in the pulp resemble those of other hard tissues. Depending on a number of poorly defined factors, surviving post-mitotic odontoblastic cells can secrete tertiary dentin, a process known as reactionary or reparative dentinogenesis, or, alternatively, perivascular progenitor cells in the pulp can be triggered to differentiate into odontoblastic-like cells under the influence of specific growth factors [6,7].

Of the numerous growth factors normally expressed during primary odontogenesis (for a review of these factors, see [8]), members of the transforming growth factor beta (TGF-beta) superfamily, including several members of the bone morphogenetic protein family (e.g. BMP-2, BMP-7), and insulin-like growth factor-1 (IGF-1) appear to play a key part in the induction of odontoblast-like cell differentiation from progenitor pulpal cells [9-12]. A number of these growth factors are incorporated into the developing dentin matrix during initial tooth formation, forming a reservoir from which they can be released following dentin breakdown.

The origin of pulpal progenitor cells remains elusive, although recent evidence suggests that they are associated with the smooth muscle cells and pericytes of pulpal blood vessels [13]. Migration of these newly proliferating stem cells to the injury site may, in part, be mediated by endothelial injury [14]. Glucocorticosteroids may also play a role in promoting differentiation of pulpal multipotential mesenchymal progenitor cells into odontoblast-like cells [15].

### B. Conventional techniques for inducing pulpal repair

Calcium hydroxide has long been the "gold standard" for pulp capping [16]. Its effectiveness at promoting dentinal bridge formation over small pulpal exposure sites is believed to be related to a combination of antimicrobial activity (attributed to high pH) and its ability to stimulate tertiary dentin formation (attributed to the release of calcium ions). Recently, mineral trioxide aggregate (MTA) has been proposed as an alternative to calcium hydroxide for pulp capping. *In vitro *[17] and *in vivo *studies [18] suggest that MTA may be more effective at inducing dental hard tissue formation than calcium hydroxide, possibly via a physicochemical reaction in which released calcium ions react with tissue phosphates to form hydroxyapatite.

### C. Research methodologies

Tooth organ culture techniques can be used for short-term *in vitro *applications. However, an animal model is needed to assess the long-term feasibility of GETE approaches to dental hard tissue regeneration because the regenerative process involves the interplay between several tightly regulated biologic systems including the host immune response, hormonal control, and poorly-defined growth factors.

Commonly used animal models for examining the effects of pulp capping agents on teeth include the dog [19], monkey [20], ferret [21], and rat [22]. Lagomorphs, such as the rabbit, have also been used [23,24]. However, both rat and rabbit teeth are continually erupting and have an open apical foramen. These two latter models have an inherent self-reparative capacity and share more similarity to human deciduous teeth and permanent teeth with immature root formation. Therefore, they are well-suited to studying the differentiation of dental progenitor cells.

The most common experimental protocol involves the creation of a mechanical pulpal exposure. This technique fails to replicate the most common clinical scenario in which the dentin-pulp complex is destroyed by bacterial-induced inflammation. Therefore, models have been developed in which pulpal inflammation is induced by the injection of lipopolysaccharide [25].

### D. Research to date

To date, attempts to regenerate lost dental hard tissue have met with mixed results.

#### a. Growth factor delivery

While intrapulpal implantation of TGF-beta1 can induce differentiation of odontoblast-like cells and reparative dentin formation in the immediate vicinity of the implanted site [26], its usefulness as a pulp capping agent is limited [27]. Application of insulin-like growth factor-1 (IGF-1) to mechanically exposed pulps appeared to reduce inflammation, preserve pulp vitality and promote pulpal repair in the rabbit [23]. *In vitro *experiments suggest that dentin matrix extract (DME), which contains a complex mixture of bioactive molecules, is capable of inducing differentiation of pulp progenitor cells into odontoblast-like cells [28]. Efforts at forming reparative dentin *in vivo *using dentin matrix extract [29-31], supraphysiologic doses of recombinant BMPs [32,22], bone sialoprotein [33] or amelogenin gene splice products [34] have resulted in either minimal dentin formation or excessive quantities of ectopic bone-like material that occlude the pulp canals. In one rat model [32], pulp capping with MTA produced significantly more dentin sialophosphoprotein (DSP), a marker of dentinoblast differentiation, compared to recombinant BMP-7. A plausible explanation for these varied results is that the delivery of a single bolus of a morphogenic protein with a short *in vivo *half-life does not provide the sustained delivery of physiologic levels of these proteins required for complete hard tissue regeneration. Moreover, it appears that higher concentrations of some growth factors may have an opposite effect, inducing apoptosis of putative progenitor cells [29].

#### b. Stem cell delivery

A number of recent studies have demonstrated that stem cells, of both dental and non-dental origin, are capable of inducing odontogenesis and regenerating dentin [35]. Human adult dental pulp contains a population of cells ("dentin pulp stem cells"; DPSCs) with stem cell-like properties such as self-renewal and the ability to differentiate into adipocytes and neural-like cells [36], but not chondroblasts [37]. Tooth-like tissues have been engineered by implanting single cell suspensions isolated from porcine third molar tooth buds seeded onto polyglycolic acid beads into the omenta of athymic rats [38]. While this preliminary research is extremely promising, one of the disadvantages of these techniques in their current state is the inability to regulate the shape and size of the regenerated tissue [39].

Deciduous teeth [40] contain a population of more immature multipotent stem cells ("stem cells from human exfoliated deciduous teeth"; SHED), that in contrast to DPSCs, are capable of forming dentin-like structures but not a complete dentin-pulp complex. Explants consisting of adult bone marrow stem cells and oral epithelium from E10.0 mouse embryos have the potential to form crude tooth-like tissues when grown in kidney capsules [41].

Supplementation of autologous tooth-derived progenitor stem cells with supraphysiologic levels of recombinant growth factors appears to hold promise for dentin/pulp regeneration. In a dog model, isolates of autologous pulp-derived cells, expanded in culture and supplemented with rhBMP-2, appear to stimulate the differentiation of odontoblasts as well as to promote new dentin formation [42].

#### c. Gene-enhanced tissue engineering for growth factor delivery

To date, only a few groups have actively investigated the use of GETE in dentin/pulp regeneration. Transfer of BMP-7 ex vivo transduced autologous dermal fibroblasts in a collagen hydrogel into an experimentally-induced ferret model of reversible pulpitis induces reparative dentinogenesis and regeneration of the dentin-pulp complex [25]. However, in this same model, *in vivo *transduction of inflamed pulpal tissue with recombinant adenovirus containing the BMP-7 cDNA was ineffective at producing dentinogenesis.

*In vivo *ultrasound-mediated delivery of BMP-11 (Growth/differentiation factor 11) cDNA to mechanically-exposed canine pulp tissue was effective at promoting significant amounts of reparative dentin formation *in vivo*, with minimal pulpal inflammation or necrosis [43]. Expression of dentin sialoprotein mRNA, a marker associated with odontoblastic differentiation, was confirmed. These findings contrast with earlier results in which gene delivery by electroporation resulted in thermally-induced pulpal necrosis [44]. *Ex vivo *transplantation of BMP-11-transfected autogenous dental pulp stem cells stimulated reparative dentin formation in the dog model [45]. These transfected dental pulp stem cells expressed markers of odontoblastic differentiation *in vitro*.

### E. Challenges and potential pitfalls

Prolonged pulpal infection will lead to severe hemodynamic changes and inflammation, compromising the vitality of the dentin-pulp complex. *In vivo *gene therapy techniques will likely only be effective for dentin regeneration/pulp capping situations in which some viable, uninfected apical pulpal tissue containing an adequate number of pulp progenitor stem cells is still present after all infected/necrotic pulpal tissue has been excavated.

*Ex vivo *approaches, in which growth factor-enhanced cells are transplanted into the tooth, might be viable alternatives for those situations in which there is substantial inflammation. Implanted cells would require a source of oxygen and nutrients to sustain viability. Therefore the local wound environment requires the ability to develop a vascular bed; either from remaining elements of the dental pulp or in the presence of a patent apical foramen. The ability of implanted cells to survive in an animal model of dental pulp exposure has been previously demonstrated [43]. Interestingly, in the myocardial injury model, transfection of bone marrow-derived stem cells with the fibroblast growth factor-2 (FGF-2) gene increases cell survival under hypoxic conditions [46]. This observation could potentially be exploited to increase the effectiveness of GETE approaches for dentin regeneration.

In addition to neovascularization, complete restoration of the dentin-pulp complex will also require regeneration of the pulpal nerve supply. The BPMs appear to play a role in stimulating nerve regeneration, while angiogenesis is regulated by VEGF.

Key questions regarding our understanding of factors regulating the dentin-pulp complex remain unanswered. For example, it is not understood how, under normal physiologic conditions, complete mineralization of the pulp is prevented, while dentin formation continues to occur at the periphery [47]. As our understanding of these signal transduction mechanisms increases, additional approaches for gene-enhanced tissue regeneration of the dentin-pulp complex will likely be developed.

## iii. Gene-based therapies for periodontal regeneration

### A. Background

The periodontal attachment comprises a heterogeneous population of tissues and cells that function, in part, to attach the tooth to the supporting alveolar bone. Additional functions include homeostasis, repair of damaged tissue and proprioception. Major components of the periodontium include the gingiva, periodontal ligament (PDL), cementum and the surrounding alveolar bone.

The word "periodontitis" literally means "inflammation around the tooth." In dentistry, periodontitis refers to a microbial-induced inflammation of the structures surrounding and supporting the teeth with resultant destruction of the attachment fibers and supporting bone that hold the teeth in the mouth. Left untreated, it can lead to tooth loss.

Periodontal disease involves a complex interaction, mediated in large part by an individual's host immune response to microbial colonization of the periodontal attachment apparatus, modified by host factors such as tobacco smoking, underlying disease states, level of plaque control and genetic susceptibility [48]. A number of studies [49] have shown an apparent causal link between genetic polymorphisms of the proinflammatory cytokine interleukin-1 (IL-1) and the severity of periodontal disease in specific populations.

It is estimated that mild periodontitis affects greater than 90% of the adult population [50]. However, attempts at determining the exact prevalence of periodontitis in adult populations are complicated by the variability in parameters examined between different researchers. Moderate to severe periodontal disease, defined loosely as periodontal attachment loss that predisposes the patient to tooth mobility and loss, affects at least 15% of adults over the age of 30 years of age [51]. In the US, the economic cost of treating and preventing periodontal disease was estimated at $14,300,000,000 in 1999 [51].

### B. Conventional techniques for periodontal repair

Currently, much of the armamentarium available to the periodontist and general dentist is focused on arresting periodontal disease progression by reducing the microbial levels in the periodontal attachment apparatus and altering the local environment to discourage reattachment of these pathogens. These techniques, which include non-surgical techniques such as scaling and root planing and surgical procedures such as open flap debridement for access and resective techniques, are designed to remove diseased tissue and promote an ideal environment for periodontal repair. The ultimate goal is to prepare an endotoxin and pathogen-free local environment that promotes reattachment to the root surface. These approaches generally result in repair, characterized by healing of the wound site by formation of an epithelial reattachment. This epithelial attachment, known as a long junctional epithelial attachment, is formed by keratinocytes that migrate into the pocket from the crevicular epithelium. The principal disadvantage of these techniques is that they represent repair of the diseased site with a non-physiologic epithelial attachment. They fail to regenerate a strong attachment between root surface and neighboring alveolar bone.

The ultimate goal of periodontal therapy remains the predicable three-dimensional repair of an intact and functional periodontal attachment that replicates its pre-disease structure. Current approaches to regenerating lost attachment have been hampered by the necessity to regenerate several tissue types: root cementum, alveolar bone and intervening periodontal ligament in a coordinated fashion.

### C. Research methodologies

Recently, several promising approaches to periodontal tissue regeneration have been developed. Proper evaluation of the clinical success rates of these different techniques has been hampered by a lack of consistency in experimental techniques used to induce periodontal defects among different groups, as well as disparities in the methods used to analyze the outcome. Proper evaluation of the validity of these techniques should ideally follow a sequential approach involving *in vitro *experiments, followed by *in vivo *confirmation in an animal model, ultimately leading to human clinical trials. The effectiveness of any periodontal regenerative approach should be evaluated *in vivo *by a combination of intraoral radiology, three-dimensional micro computed tomography (microCT), and histologic/immunohistochemical techniques [52].

The most popular animal models used for the assessment of periodontal regenerative protocols involve [53] ligature-induced periodontal defects in the non-human primates (especially the cynomolgus and rhesus monkeys, which share marked similarity to the human periodontium in terms of structure, plaque flora, and inflammatory infiltrate), and beagle dogs (which have a different microflora and much faster bone turnover rate compared to humans).

Obvious ethical issues preclude the *en bloc *harvesting of tooth, periodontal ligament attachment and supporting alveolar bone that would be required for microCT and histologic evaluation in human clinical trials [54]. Therefore, by necessity, the assessment of efficacy in clinical trials requires a combination of intraoral radiographic evaluation and clinical assessment of attachment gain. Attempts to statistically analyze the effectiveness of these techniques has been hampered by the observation that some subpopulations appear to respond better to treatment than others.

Identification of the type of cementum produced is also a vital component of the evaluation of any successful periodontal regenerative procedure. There are four principal types of cementum [55,56]. Acellular extrinsic fiber cementum (AEFC) contains extrinsic fibers (Sharpey's fibers), laid down by PDL, and serves to anchor the root to the PDL. This type of cementum should be viewed as the "gold standard" in periodontal regeneration. Cellular mixed stratified cementum (CMSC), found in the apical and furcation regions of molars areas, consists of a mixture of AEFC and cellular intrinsic fiber cementum. Cellular intrinsic fiber cementum, known as repair cementum, is typically seen in association with reparation of resorption defects. It lacks Sharpey's fibers and therefore has no direct role in tooth attachment. Acellular afibrillar cementum, also called coronal cementum, is found only on enamel at the cementoenamel junction. Its precise function is unknown.

### D. Research to date

#### a. Bone replacement grafting

Bone replacement grafting techniques, principally using autogeneic and allogeneic grafts, are widely used in the clinical setting. Evidence suggests that autogenously harvested cancellous bone grafts, obtained from iliac crest, the maxillary tuberosity or healing tooth extraction sockets, are capable of producing statistically significant bone fill. The limited ability of cancellous bone grafts to repair and/or regenerate bone and periodontal attachment involves at least three separate but distinct mechanisms: the ability of bone to act as a biocompatible scaffold, the presence of specific growth factors within the bone matrix, and, depending on the source of graft material employed, the existence of a small population of bone marrow stem cells that may be capable of differentiating into the specific cells required for bone/periodontal regeneration. Disadvantages with the use of fresh iliac crest grafts include root resorption and the requirement for a second surgical site. Moreover, histological evidence of true periodontal regeneration in these cases has been limited [57]. In many instances, alveolar bone regeneration is seen in association with the formation of a long junctional epithelium, representing periodontal repair and not true regeneration.

Limited human clinical studies have demonstrated histological evidence of periodontal regeneration, primarily limited to the base of the defect, through the use of decalcified freeze-dried allogeneic bone (DFDB) grafts obtained from commercial tissue banks [58]. Drawbacks include the possibility of eliciting a host immune response, the risk of disease transmission, and the apparent wide variability in the concentration of bone and periodontal-inductive agents (and hence biological activity) between different preparations.

#### b. Guided tissue regeneration

Guided tissue regeneration (GTR) is an approach to regaining periodontal attachment loss involving the surgical implantation of a cell-impermeable barrier between detoxified root surface and the crevicular epithelium. The goal is to retard the migration of crevicular epithelium into the space between the newly prepared root surface and the neighboring alveolar bone, thereby avoiding the formation of a long junctional epithelium. Presumably this affords time for the selective repopulation of the root surface by cells from within the PDL space. This approach may also permit putative progenitor cells within the periodontal defect to differentiate into the specific cell types required for the regeneration of a functional periodontal attachment under the stimulus of poorly defined signaling/growth factors. A Cochrane review of published studies [59] suggests that guided tissue regeneration can be effective at regenerating periodontal attachment to a limited extent, but the overall response rate is unpredictable. Differences may be related to variations in the numbers of putative progenitor stem cells and the concentrations of appropriate signaling factors in the periodontal defect site between patients.

#### c. Growth factor delivery

A number of approaches for periodontal regeneration that are currently being investigated involve direct delivery of growth factors. The scientific basis behind these newer periodontal regenerative approaches lies in part with the existence of putative precursor cells within the vicinity of the periodontal attachment. These cells are believed to be capable of differentiating into the more specialized cell types required for the reconstruction of a functioning periodontal attachment apparatus (osteoblasts, cementoblasts, fibroblasts), under the influence of specific growth factors. Putative growth factors common to both cementum and bone include [55] members of the TGF-beta superfamily, such as the BMPs, as well as IGF-I and IGF-II, platelet-derived growth factors (PDGFs), epidermal growth factor (EGF), and the fibroblast growth factors (FGFs). In addition, cementum-derived growth factor (CGF), an isoform of IGF-I, appears to be cementum-specific [60]. These growth factors can be further subdivided into those that stimulate osteogenesis (e.g. bone morphogenetic proteins), those that promote cellular differentiation (e.g. platelet-derived growth factor) and angiogenesis (e.g. vascular endothelial growth factor; [61]), and those that regulate the epithelial mesenchymal interactions involved in initial tooth formation (e.g. Embdogain™).

Emdogain™ (Strauman AG, Basel, Switzerland), a mixture of enamel matrix proteins, primarily amelogenins, isolated from developing porcine teeth, has been approved by the U.S. Food and Drug Administration (FDA) for regeneration of angular intrabony periodontal defects. Although the mode of function is not known, the proposed mechanism behind using enamel matrix proteins is that these proteins are believed to be involved in forming the periodontal attachment apparatus during initial tooth development. The addition of these proteins to periodontal defect sites may be effective at promoting periodontal regeneration by recapitulating the environment during initial tooth attachment. Recent studies [62] have shown that Emdogain™ contains both TGF-beta and BMP growth factors, that may contribute to its clinical effectiveness. A systematic review of published clinical trials [63] suggests that Emdogain™ affords results similar to those seen with the use of GTR.

Platelet-rich plasma (PRP) is a component of autologous whole blood isolated following the centrifugation of the plasma. PRP acts as a source of growth factors including PDGF and TGF-beta, both of which appear to be critical growth factors involved in periodontal regeneration. The availability of several commercial kits to isolate PRP at chairside has contributed to its increasing popularity among clinicians. Preliminary studies [64,65] suggest that while PRP may have limited effectiveness at promoting periodontal regeneration, results with PRP for bone regeneration have been contradictory [66]. Wide differences in the concentration of growth factors between different preparations and between different patients may account for some of the disparate results. Large scale human studies are required before this technique can be recommended for routine use.

Recombinant human BMP-2 (rhBMP-2) and rhBMP-7 have been extensively investigated as to their ability to regenerate periodontal structures. Ankylosis has been observed in some models of periodontal regeneration, although results have been conflicting. In furcation defects, BMP-2 caused ankylosis at the cementum-enamel junction in a dog model [67], whereas, in baboons, BMP-7 did not [68]. These differences may be related, in part, to the animal models, type of defect created, whether the treated teeth are in occlusion, as well as the carriers used [9]. Other growth factors employed with varying success have included PDGF +/- IGF-I [69,70], FGF-2 [71], TGF-beta1 [72], and brain-derived neurotrophic factor [73]. Several reviews detailing the strengths and weaknesses of these different growth factors for periodontal regeneration have been written [74-76].

As our understanding of the different growth factors involved in dental development increases, the number of potential therapeutic agents will likewise grow. However, the principal drawback with these techniques is that these growth factors, which generally have a short *in vivo *half-life, are delivered as a single non-physiologic bolus in most techniques. Development of controlled-release delivery approaches has the potential to significantly increase their clinical effectiveness [77].

#### c. Cell delivery

The exact source of periodontal precursor cells has yet to be determined, although it is believed that they are most likely located within the PDL. A population of multipotent postnatal stem cells have been isolated from human PDL (PDLSCs) that are capable of generating cementum/PDL-like structures when transplanted into immunodeficient rats [78]. These PDLSCs expressed the cell surface marker STRO-1, an early mesenchymal stem cell marker, and have the potential to differentiate into fat cells following induction with an adipogenic cocktail. These adult stem cells can be recovered from cryopreserved solid tissue isolated from the periodontal ligament of extracted third molars and are likewise able to generate cementum and periodontal ligament-like structures *in vivo *[79].

The use of bone marrow-derived stem cells for periodontal regeneration has also been evaluated. Preliminary results involving 7 patients who received autologous iliac crest bone marrow cells demonstrated some gain of clinical attachment [80].

#### d. Gene-enhanced periodontal regeneration

The goal of gene-enhanced periodontal regeneration is to reclaim the lost regenerative capacity within the PDL space. While GETE can be used in conjunction with stem cells, this technique has the greatest potential if it can be adapted for use with easily harvestable fully mature cells (e.g. gingival fibroblasts, periodontal ligament fibroblasts). These cells are then genetically-enhanced to express growth factors that are involved in the initial formation of both dental and periodontal attachment tissues. In short, this approach is intended to mimic the normal biological process that occurs as these tissues are formed early in development. More specifically, transient morphogen stimulation, combined with local cues in the wound environment, primes progenitor cells within the periodontal ligament to differentiate into the specific cells required for the production of *root cementum, alveolar bone and PDL fibers *in a *coordinated *fashion.

GETE for periodontal regeneration is still in its infancy. A couple of preliminary studies have confirmed that this is a promising approach. Syngeneic dermal fibroblasts transduced *ex vivo *with an adenoviral vector expressing BMP-7 (Ad-BMP-7) in a gelatin carrier were implanted into submerged, surgically-created periodontal-alveolar bone defects in the rat [81]. Significant bridging of the alveolar defect was seen in conjunction with new cementum formation and fibrous connective tissue attachment. Interestingly, new bone formation occurred through a process of endochondral ossification. Direct *in vivo *transfer of PDGF-B stimulated both alveolar bone and cementum regeneration in a rat acute periodontitis model [82].

### E. Challenges and potential pitfalls

It can be seen from the above discussion that successful regeneration requires the sequential coordination of a number of tightly-related processes. First, endotoxin contamination of the root surface needs to be reduced. Then, progenitor cells within the PDL need to differentiate into several cell types (i.e. osteoblasts, cementoblasts, fibroblasts, and endothelial cells). These cells must subsequently synthesize and release their specific cellular products in a coordinated and sequential manner to ultimately regenerate AEFC and Sharpey's fibers, connecting the root surface to the alveolar bone and thus regenerating a functional periodontal ligament.

In the future, the incorporation of biomimetic motifs into matrices (e.g. addition of cementum-derived attachment protein, a cementum-derived protein that appears to promote adhesion of mineral-forming mesenchymal cells to root cementum; [83] holds significant potential for increasing the success rate of periodontal regenerative protocols.

A number of unknowns remain to be answered before ideal conditions for periodontal regeneration can be developed. For example, the specific factors that induce differentiation along cementoblast lineage, as well as the origin of cementoblasts, are not known [55].

## v. Practical considerations and future prospects

While it is anticipated that in the future, gene-enhanced tissue engineering approaches will afford great potential for both dentin-pulp and periodontal regeneration, this approach would currently face significant regulatory hurdles prior to government approval. With the continued development of improved methods for gene delivery to cells as well as advances in our knowledge of the molecular basis of tooth formation and periodontal homeostasis, it is reasonable to anticipate that a simple chairside protocol could be developed in the future. This might involve either the direct delivery of the DNA of interest to the pulpal/periodontal tissue, or the isolation of a small amount of gingival tissue from the patient, transduction/transfection of the DNA at chairside, and reimplantion of the gene-enhanced cells into the tooth or periodontal ligament space.

## Competing interests

The author(s) declare that they have no competing interests.

## Authors' contributions

Both authors contributed equally.
